# Crisis Communication during COVID-19: English, French, Portuguese, and Spanish Discourse of AstraZeneca Vaccine and Omicron Variant on Social Media

**DOI:** 10.3390/vaccines11061100

**Published:** 2023-06-15

**Authors:** Daniel Catalan-Matamoros, Ignacio Prieto-Sanchez, Andrea Langbecker

**Affiliations:** Medialab Research Group, Department of Communication and Media Studies, Madrid University Carlos III, 28903 Getafe, Spain; dacatala@hum.uc3m.es (D.C.-M.); ignpriet@pa.uc3m.es (I.P.-S.)

**Keywords:** vaccination, Omicron, AstraZeneca, Twitter, social media, crisis communication, global discourse

## Abstract

Social media have been the arena of different types of discourse during the COVID-19 pandemic. We aim to characterize public discourse during health crises in different international communities. Using Tweetpy and keywords related to the research, we collected 3,748,302 posts from the English, French, Portuguese, and Spanish Twitter communities related to two crises during the pandemic: (a) the AstraZeneca COVID-19 vaccine, and (b) the Omicron variant. In relation to AstraZeneca, ‘blood clot’ was the main focus of public discourse. Using quantitative classifications and natural language processing algorithms, results are obtained for each language. The English and French discourse focused more on “death”, and the most negative sentiment was generated by the French community. The Portuguese discourse was the only one to make a direct reference to a politician, the former Brazilian President Bolsonaro. In the Omicron crisis, the public discourse mainly focused on infection cases follow-up and the number of deaths, showing a closer public discourse to the actual risk. The public discourse during health crises might lead to different behaviours. While public discourse on AstraZeneca might contribute as a barrier for preventive measures by increasing vaccine hesitancy, the Omicron discourse could lead to more preventive behaviours by the public, such as the use of masks. This paper broadens the scope of crisis communication by revealing social media’s role in the constructs of public discourse.

## 1. Introduction

A total of 6,606,624 people have already died as a result of the SARS-CoV-2 epidemic globally [1]. Several significant events have taken place since the WHO declared the COVID-19 pandemic in March 2020, generating a huge amount of global public anxiety, fear, and uncertainty. Since then, the majority of governments came to the conclusion that they should act urgently to address the crisis and eventually be allowed to take control of this serious epidemic. It is well known that crisis communication plans can play a crucially important role in the long-term prevention and mitigation of pandemics by reducing fear and uncertainty, encouraging public adherence to mitigation measures, softening the burden, and strengthening the effectiveness of patient care [2]. With these regards, government officials have used a variety of crisis communication techniques to convince the general public to follow specific guidelines for preventing the virus’ spread and so reduce people’s fears and worries [3].

Large-scale social crises, such as the COVID-19 pandemic, are followed in contemporary democracies by amplified public discourse regarding the most correct policies and response strategies [4]. Digital media magnifies these challenging situations by enabling average citizens to express their opinions and engage in public discourse while news organizations, journalists, or authorities lose their unique position as gatekeepers and information brokers [5]. Twitter data, for example, opens up new avenues for studying these dynamics. Digital trace data from social media provide valuable opportunities to analyze communication patterns during crises. As more and more mediated communication occurs on digital platforms, data from those platforms is becoming a useful tool for objectively analyzing public discourse [6]. Comprehensive research on how crises influence public discourse in different communities, however, is still limited [4]. Therefore, this article will investigate the impact of COVID-19 on public discourse in the Twitter-sphere with regard to four communities according to language (English, French, Spanish, and Portuguese) focusing on two specific crises that took place during the pandemic: (a) the AstraZeneca COVID-19 vaccine, and (b) the Omicron variant.

During this pandemic, many people turned to social media platforms for information or to express their opinions on significant developments. As a result, the findings of this study provide significant data that shed light on the most common problems and arguments witnessed on Twitter across different international communities. Furthermore, these findings can serve as a guideline for health communication strategies, allowing governments to implement successful communication methods that take into account the complexities of social networks in a variety of conditions. Furthermore, these findings help to broaden our understanding of this phenomenon, developing a global perspective.

### Literature Review

During the pandemic, the issue of vaccines attracted a considerable amount of public attention. On 8 December 2020, immunization as a strategy to lower the number of fatalities and hospitalizations started in the United Kingdom and expanded internationally with varying vaccination rates, as shown by differences in vaccine availability. We have a broad choice of vaccines on the market now thanks to the fast development and production of COVID-19 vaccines [7,8]. In the case of the AstraZeneca/Oxford vaccine, it was approved by the European Medicines Agency (EMA) in January 2021. However, reports of rare blood clotting disorders (thrombosis with thrombocytopenia syndrome) related to this immunization, in March 2021, have led many European Union countries to choose to temporarily suspend its use, even though the World Health Organization (World Health Organization 2019a) and EMA took a position in favor of its safety, stressing that the benefits outweighed the risks. However, this event might have had an impact on people’s adherence to vaccines, which was crucial at a time when vaccination programs were being initiated. With 185 countries now using it, this vaccination is currently the most popular worldwide [9].

The emergence of the new Omicron variant, which was initially discovered in South Africa in November 2021, also had an impact on pandemic management and raised public discourse. It generated a large amount of fear at the time since it was not known if it was as dangerous as the Delta variant. The degree of the disease’s transmission, and the possibility that this variety might have a more severe clinical presentation, were both unknown [10]. Today, it is established that the risk of infection with omicron is far higher than that of the earlier subvariants, which are mostly present globally and are highly infectious [11].

In situations such as these, health authorities and scientists identify and analyze the real risk. Based on this evaluation, they design strategies and make decisions. On the other hand, people’s perceptions of risk in relation to these events do not always reflect actual risk. Understanding this viewpoint is essential because it influences people’s willingness to accept or refuse the preventive measures being taken to control the pandemic, such as vaccination, mask usage, and social isolation [12]. Risk perception is subjective, and may be related to fear, beliefs, and previous experiences. It can affect personal relationships, trust in authorities and the media, and scientific uncertainty, among other issues. People’s responses to it will be influenced by their perception of risk, which may contribute to, for instance, vaccine hesitancy [13]. This phenomenon occurs when the person has the possibility to be vaccinated but does not want to or refuses to take it [14]; this is considered by the WHO as one of the top ten threats to global public health [15]. However, social media play a key role in favoring the increased reluctance towards vaccination and other preventive measures against coronavirus to disseminate false and alarmist content [13,16,17]. This kind of information is generated by the anti-vaccine lobby, which uses social media as an effective tool for spreading their beliefs rapidly [18].

Twitter, which is still distinguished by its capacity to generate noise rapidly, is one of the social media platforms that are relatively commonly used to propagate misinformation [19]. Created in 2006, it is a microblog characterized by short texts (up to 280 characters) in which users can also send photos or videos, being sent 500 million tweets per day. It currently has 436 million active users; 70.4% of them are men, while only 29.6% are women. Formed by users with a more mature profile than those of TikTok and Snapchat, the majority are between 25 and 34 years old (38.5%). In terms of educational level, most have a college degree or higher (33%) [20,21]. Additionally, Twitter is acknowledged as the social media platform with the most health-related content [22]. In this sense, this social network can be useful to investigate the public discourse related to these two crises that impacted the course of the pandemic, the AstraZeneca-related thrombus cases, and the circulation of the new omicron variant. There are many studies that have investigated the public discourse about COVID-19 vaccines on Twitter [23,24,25,26], but few of them did so specifically on these two specific crises. Marcec and Likic [27], for example, have investigated sentiments towards AstraZeneca/Oxford, Pfizer/BioNTech, and Moderna vaccines on English posts on Twitter and Jemielniak and Krempovych [17], related to misinformation and fear about the AstraZeneca vaccine also on this same social network. The first study [27] has found that positive sentiments towards AstraZeneca declined over time with a significant drop when comparing December 2020 to March 2021. The sentiment related to this vaccine turned slightly negative in March 2021. To the authors, one of the reasons could be probably related to the thrombotic events that happened in this period. This fact may collaborate to increase vaccine hesitancy. In the case of the second study [17], the circulation of misinformation not only came from the anti-vaxxer movement but was also carried out by professional sources. The authors highlight the negative campaign related to this vaccine on Twitter which can be provoked by political and economic issues.

In relation to Omicron, Mahyoob et al. [28] investigated sentiments towards this variant on Twitter, and Thakur and Han [29] analyzed tweets related to opinions and experiences about this new variant. The existing literature on social media and the crises under investigation (Astra-Zeneca vaccine and Omicron variant) primarily examines posts published in English, leaving significant gaps in our understanding of the public discourse on social networks. By focusing solely on one language, we limit our comprehension of the broader public debate surrounding these issues. To address this limitation, our study aims to expand this perspective by conducting a comparative analysis across diverse communities using a substantial volume of data. This comparative approach allows us to identify both similarities and differences that have shaped the discussions surrounding these two crucial crises during the pandemic period.

## 2. Materials and Methods

The article follows a quantitative methodology based on big data analysis techniques. After building a large-scale Twitter dataset, text filtering has been applied to remove unwanted elements in tweets. Numpy and Pandas APIs were used to process the large databases due to their widespread use and high performance. Thanks to them, filtering and transformations that were necessary for the two quantitative analysis methodologies can be efficiently applied.

**Figure 1:**
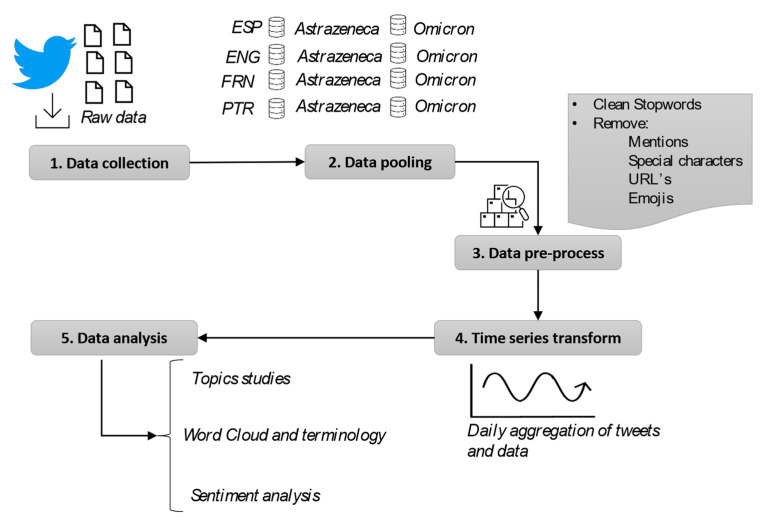
Summary of research steps.

Figure 1 shows the collection and processes performed on the data to achieve the results. Three different methodologies of analysis were applied to the data.

### 2.1. Data Collection and Pooling

For the development of the study, eight different databases were built. The tweets were separated by specific crisis and language. Using the Python Twitter API with the research license, we have been requesting data during the Omicron and AstraZeneca crisis periods. The data collection process involved utilizing the Tweetpy library to make requests related to various topics of interest, such as COVID-19, Vaccines, AstraZeneca, Omicron, and more. These requests enabled us to retrieve tweets that specifically mentioned these topics, along with other parameters of interest such as dates or retweets. The collected tweets were then compiled into extensive databases, which were subsequently organized and analyzed using Nvivo11 in conjunction with Python.

Table 1 shows the volume of each database and the period covered by each one. Thanks to the Twitter function, and chronological timeline request function, data distribution based on user activity was guaranteed. The periods with a higher volume of tweets per day are due to hot spots caused by a major event or controversy.

A total of 2,494,335 AstraZeneca and 1,253,967 Omicron-related tweets were collected and analyzed. The total size of the study was 3,748,302 tweets. Data were obtained for four languages representing different groups. Since France and Brazil make up nearly all of the weight in their datasets, the data for French and Portuguese were not very dispersed, which means most of the tweets in the database come from nearby geographical locations, even from the same country. However, the data for Spanish and English were more dispersed and come from various geographical areas. The groups represent the English, French, Portuguese, and Spanish-speaking communities. This study’s time frame covers a number of controversies and debates that accompanied both crises. Due to adverse reactions throughout the vaccination process, AstraZeneca’s initial analysis period was determined to be the most active phase. When it comes to Omicron, the behavior is similar, and the beginning of the listening corresponds with the appearance of the variant. The dates for tweet collection were carefully selected to align with the peak periods of activity surrounding these crises. The chosen starting dates correspond to the moments when these issues gained significant attention and sparked controversy within public opinion.

### 2.2. Data Pre-Processed

Using NVivo software, the data were grouped by themes and languages, moving from independent daily files to completed datasets. After organizing all tweets with the corresponding topic and language, the content of the tweets were filtered and transformed to increase the efficiency of the analysis, the final dimension of each dataset is shown in Table 2. Due to the nature of the data, the entries correspond to messages of a generally informal register. Therefore, the use of emojis, capital letters, and other unwanted elements that make the analysis difficult is frequent. Since the analysis techniques are automatic, it is necessary to ensure that the format is correct to increase the reliability of the results.

### 2.3. Data Analysis and Time Series

After obtaining the filtered data without unwanted elements, they are analyzed using different methodologies. Three different lines of work are carried out for each database: word prevalence, topics studied, and sentiment analysis. The combination of these three forms offers a plethora of information to understand how the English, French, Spanish, and Portuguese communities perceive risk.

The word prevalence is presented in a table according to the language. This allows us to observe whether one community makes more references to health organizations, side effects, or symptomatology while another focuses on polemics or misinformation.

The topics studied are focused to identify the presence of specific themes in the datasets. The themes were selected to compare the importance given by each community to different aspects that have had a major impact on the perception of COVID-19 risk. Therefore, to filter each dataset, a terminology study was carried out to find out the references most frequently used by Twitter users to talk about these concepts. Table 2 and Table 3 show the terms used for filtering the topics in each dataset. In comparing the four different languages, the filtered concepts are the same in all languages. For AstraZeneca, the importance of blood clots, side effects, anti-vaccine, number of deaths, and references to lies were quantified by this analysis. For Omicron, masks, infections, deaths, and risk were evaluated.

For the Sentiment Analysis, it was necessary to count and construct new variables in a format based on chronology. For the interest of the study, the sentiment on the social network had to be represented in a temporal format, identifying the presence of negative opinions depending on the periods. Therefore, the Sentiment Analysis is divided into two parts, the evaluation of each tweet and the temporal grouping of the categories (negative, positive, and neutral). For the individual classification of each tweet, techniques based on lemmatization are used to obtain whether the message has a positive, negative, or neutral predominance. Using the Natural Language Toolkit (NLTK) library, an open-source API that allows us to process natural language and obtain a weighting for each sentiment. In this way, a weight is obtained for each sentiment and the highest one has been selected. After rating each tweet with sentiment, they are first grouped by days. Then the number of daily tweets for each sentiment is counted. This results in three different curves representing the number of positive, negative, and neutral tweets per day. Graphically represented, the evolution of the trend can be observed by peaks or troughs. Finally, connecting the study of themes and word clouds with sentiment allows us to make a complete description of each community, being able to identify differences between them.

### 2.4. Ethical Requirements

To comply with the ethical requirements, this study lays under the PredCOV project “Multi-source and multi-method prediction to support COVID-19 policy decision-making” which has been approved by the University Ethics Committee of the Madrid University Carlos III under the CEI22_05 ID protocol.

## 3. Results

### 3.1. The AstraZeneca Crisis

#### 3.1.1. Topics

Thematic analysis is a method used to assess the prominence of various concepts related to language use. This technique can reveal how frequently key concepts, such as fear of side effects, awareness of preventive measures, mask use, and COVID-19 case monitoring, appear within different communities. In Table 4, we present the results of our search for tweets related to the AstraZeneca crisis. The table displays the number of tweets found for each topic, along with a normalized value that compares the number of tweets to the maximum number of tweets mentioned in other languages. This value indicates the relative weight of each topic compared to the others. A value of 1 indicates that the language had the highest number of tweets mentioning that topic, while a value of 0 means that there were no references to that term. Values that are far from 1 indicate that a topic had little relevance or weight compared to the others. Conversely, values close to 1 indicate that a topic had a higher number of mentions and was more relevant to the debate. The final column in the table represents the maximum percentage of tweets related to each topic, calculated as a percentage of the total number of tweets for that topic. This percentage is based on the language used to normalize the data. The higher the percentage, the more weight the topic has in the public discourse. For the filtered terms we analyzed, the percentages were 13.77%, 3.28%, 1.54%, 10.46%, and 2.52%.

A blood clot is one of the most frequently used terms during the vaccination process. This first term appears in all four languages, with different weights. For French (1), Portuguese (0.23), and Spanish (0.21), it represents the topic with the greatest weight in Twitter posts. For Spanish and Portuguese, the presence of the topic is lower than the average value. For English tweets, a higher value close to 0.5 is identified but the most striking case is observed in the French tweets, where the value is much higher than the rest of the languages. It appears in 61,176 French tweets, being mentioned in almost 14% of the posts referring to AstraZeneca for this language.

Across all languages, the average frequency of the term ‘side effects’ is 1.95%. This term is more scientific in nature, so its lower frequency is expected when compared to more colloquial terms such as ‘deaths’ or ‘thrombus’. The French-speaking population stands out with a weight almost twice as high as that of the other languages. The term ‘Anti-Vax’ is commonly used on Twitter to refer to people who are against vaccination. However, references to this term are scarce in all datasets except for the English-speaking community, where it appears in 1.54% of the posts. This concept of belonging to an anti-vaccine group or lobby is not as prevalent in other languages. In fact, this term has very little weight in tweets written in languages other than English.

The frequency of references to ‘deaths’ is an important indicator of public discourse and risk perception. The English-speaking community has the highest frequency of mentions (1) of this term, closely followed by French (0.88). These two communities use this term with high frequency in their publications. In contrast, Spanish (0.18) and Portuguese (0.15) have substantially lower values, indicating that references to ‘deaths’ are much less common in tweets written in these languages. On the other hand, references to ‘lies’ and ‘fake news’ were more frequent in English tweets.

#### 3.1.2. Words

Table 5 presents the quantified results for word prevalence in AstraZeneca-related tweets. To enhance understanding, the elements in the table have been grouped and translated. The original version contained language-specific terms, as described in the methodology. Proper nouns have been left unchanged.

The AstraZeneca crisis has generated a significant amount of discussion across all languages, with common terms including vaccination, dosage, and the names of pharmaceutical companies involved. Table 1 displays the frequency of terms present in the word clouds for each of the four languages studied. In Portuguese, official health agencies such as Covax (34,784), Butantan (22,369), and WHO (20,663) are highly referenced, along with Brazil (36,681) and its president Bolsonaro (27,320). Additionally, the Indian pharmaceutical company Bharat Biotech (19,003) is mentioned in the context of Brazil’s national vaccination program. French tweets feature a high number of references to the Moderna vaccine (96,172), France (32,111), and thrombosis (45,641), along with the terms ‘injection’ (20,656) and ‘effects’ (21,871), which are related to the vaccination process. In Spanish tweets, there is a significant focus on the vaccination process and the pharmaceutical industries involved, with frequent references to ‘vaccines’ (398,072), ‘vaccination’ (83,869), and ‘vaccinated’ (19,706). However, specific terms such as ‘side effects’ or ‘thrombosis’ are not as prevalent in the Spanish-speaking community compared to the other languages. In the English-speaking community, vaccine-related terms such as ‘vaccine’ (499,232), ‘vaccination’ (45,971), and ‘booster’ (30,813) are frequently mentioned, along with related terms such as ‘jab’ (58,929), ‘India’ (60,031), and ‘UK’ (44,872), indicating a high level of focus on the vaccination process.

#### 3.1.3. Sentiment Analysis

For AstraZeneca Tweets, a similar trend is observed across all four communities. Activity on the topic peaks at the beginning of the data collection around April 2021 and starts to decrease until the beginning of the summer. During this seasonal period, the topic was much less relevant. The theme increases again around November 2021 showing relative peaks and troughs. This period is more stable in some cases than in others.

**Figure 2:**
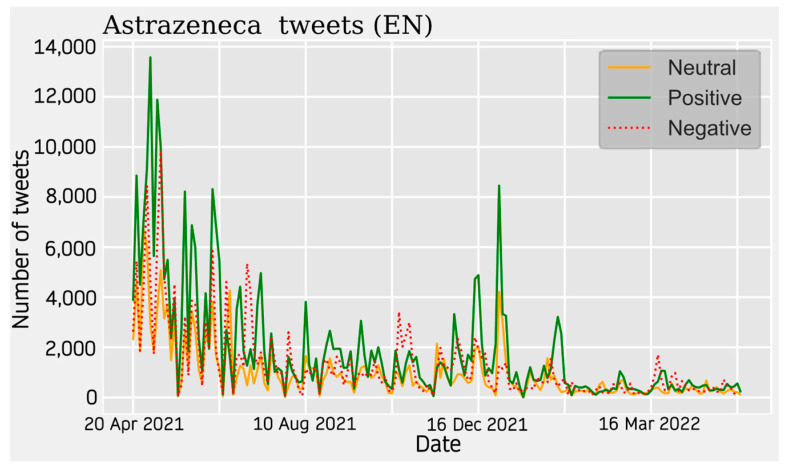
English Sentiment Analysis. The line graphs represent the number of daily tweets observed for each sentiment.

**Figure 3:**
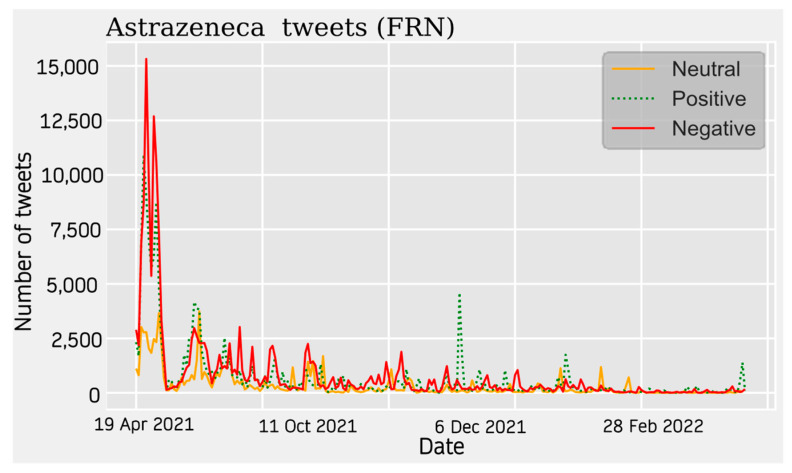
French Sentiment Analysis. The line graphs represent the number of daily tweets observed for each sentiment.

**Figure 4:**
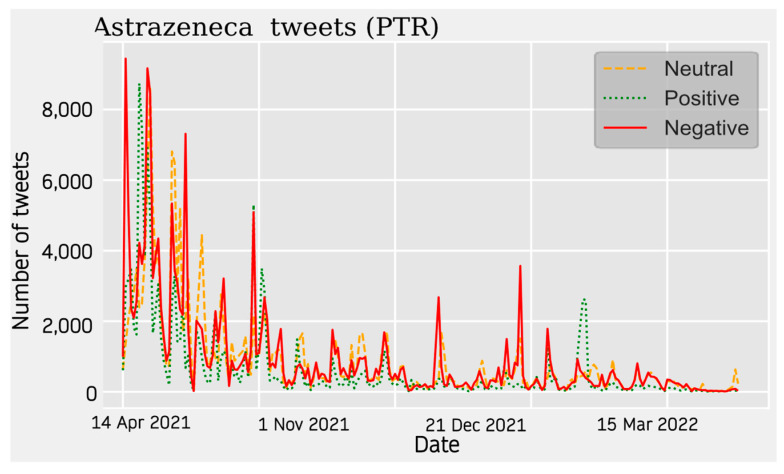
Portuguese Sentiment Analysis. The line graphs represent the number of daily tweets observed for each sentiment.

**Figure 5:**
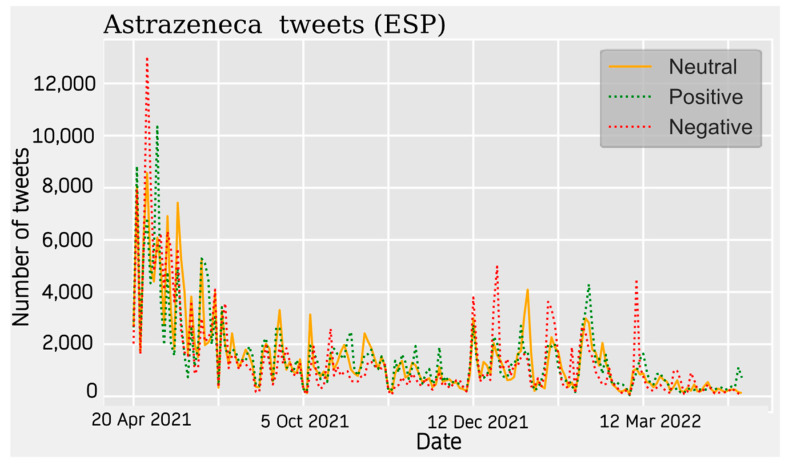
Spanish Sentiment Analysis. The line graphs represent the number of daily tweets observed for each sentiment.

**Figure 6:**
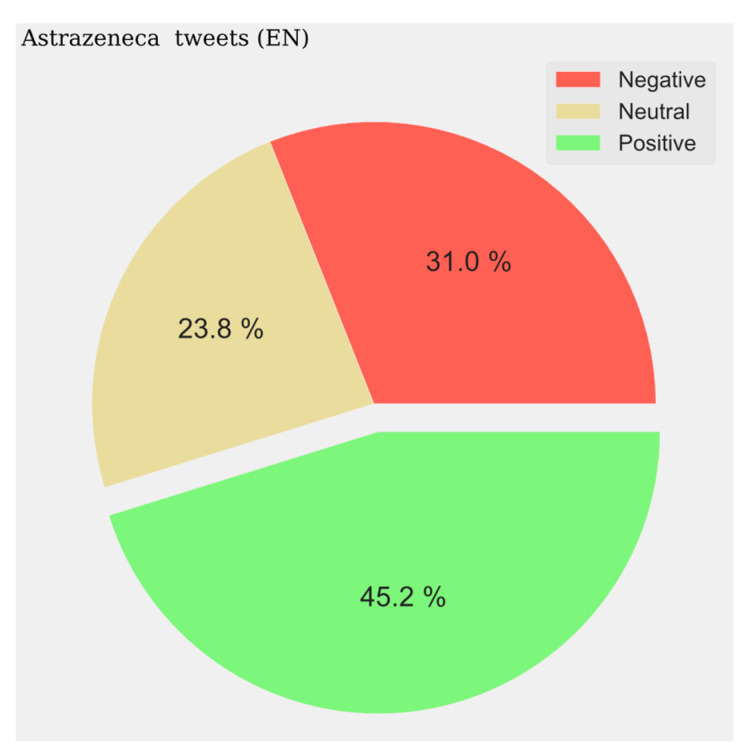
English Sentiment Analysis.

**Figure 7:**
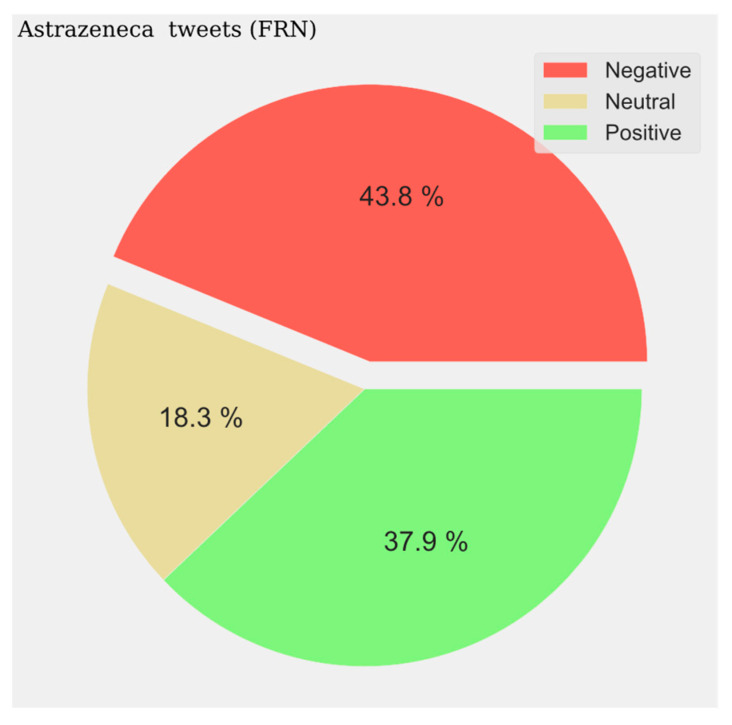
French Sentiment Analysis.

**Figure 8:**
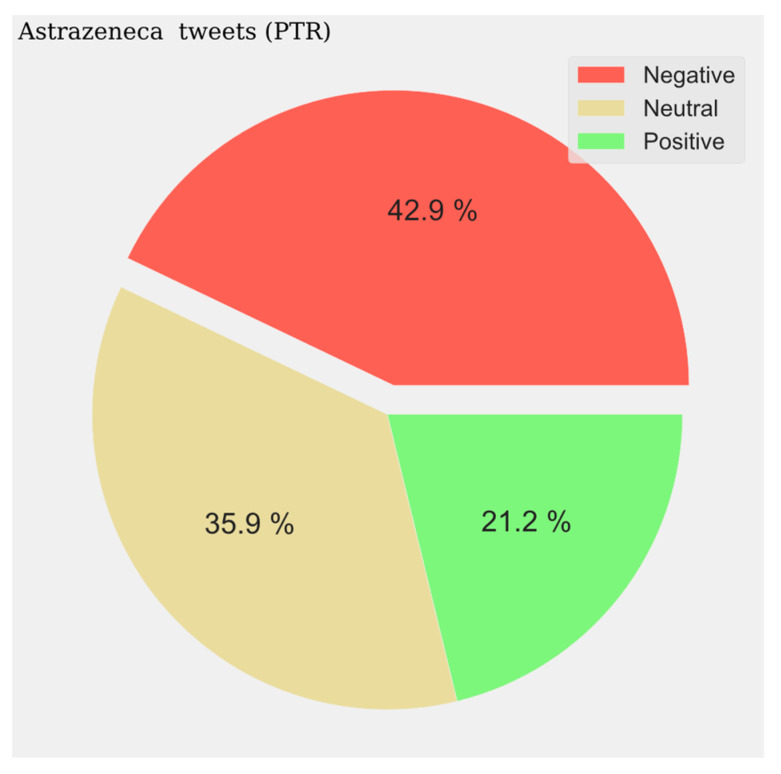
Portuguese Sentiment Analysis.

**Figure 9:**
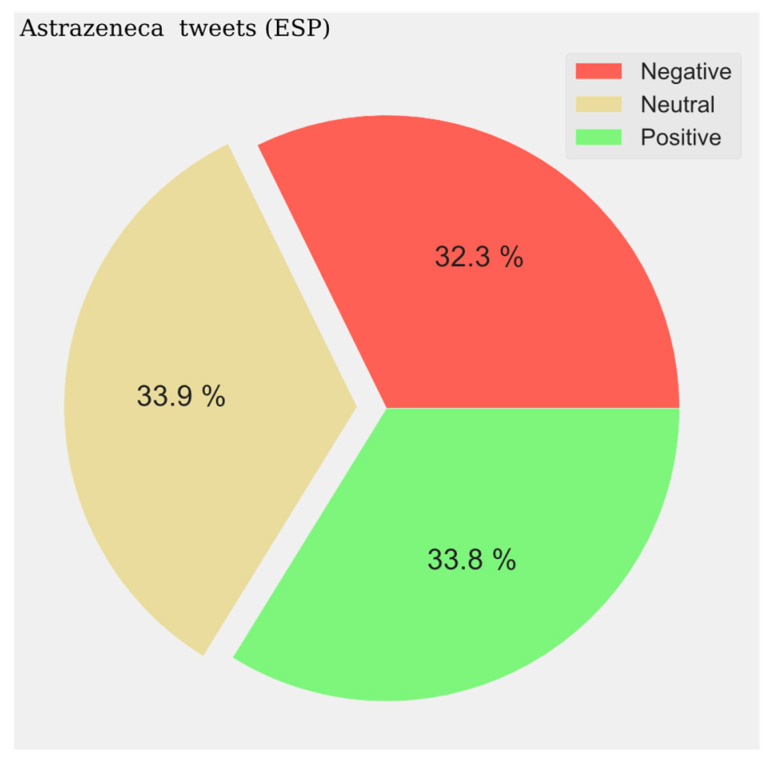
Spanish Sentiment Analysis.

With respect to the sentiment results, a solid line shows the most predominant sentiment in each dataset. Negative sentiment is the most present in the Portuguese and French communities, while for English and Spanish, it is the positive and neutral ones that are the most prevalent (Figure 2, Figure 3, Figure 4 and Figure 5). The absolute maximum of the graphs for Portuguese and French shows a clear negative predominance, more accentuated for the latter. For English posts, the polarity is more evenly distributed with a predominance of positive sentiment, while for Spanish, the sentiment is balanced. For the Spanish tweets, there are relative peaks of a negative nature from November 2021 onwards. For the French database, there is a positive relative peak in the same period. Regarding the pie charts, (Figure 6, Figure 7, Figure 8 and Figure 9) the high presence of negative opinion is observed in the French (43.8%) and Portuguese (42.9%) tweets.

### 3.2. The Omicron Crisis

#### 3.2.1. Topics

Table 6 shows the results for the Omicron data. The filtered themes have been quite present in the datasets reaching a maximum of 6.31%, 25.60%, 8.73%, and 4.25%. The ‘mask’ topic is present in all four languages studied. The highest presence was found in English tweets (1), where it was mentioned in almost 7% of tweets. In Portuguese, it has a very similar value (0.9), representing almost double the Spanish value. Masks have been one of the main measures applied to counter the virus. Its use has evolved according to the different periods and the severity of the situation.

The cases and infection related-topics have had the greatest impact on the discourse. It reaches 25.60% for the maximum value and provides a great rate of monitoring of the crisis by each community. The highest values are found for Spanish and Portuguese, which are very similar, with a high presence in their tweets. For the French and English data, the values are lower, around 0.75. This theme is closely related to risk perception and fear towards the Omicron variant. With reference to the number of deaths, we found a very atypical low value for tweets in Portuguese (0.40), which is very different from the rest of the languages.

#### 3.2.2. Words

Table 7 presents the results of the word analysis related to the Omicron crisis in each language community. The term ‘variant’ is present in all four communities, along with references to the virus and the number of cases. However, in the Portuguese tweets, there are additional references to ‘children’ (16,925), ‘immunity’ (17,436), and ‘flu’ (10,422), which are rare in the other language sets. It is noteworthy that there is a significant reference to Africa (11,361) in these tweets. Moving on to the French community, the discourse includes references to the ‘Delta’ variant (27,259), ‘France’ (27,443), ‘measures’ (16,607), and ‘children’ (14,311). Both in Portuguese and French discourses, the impact of Omicron on children is a prevalent theme when compared to the rest of the population.

In the case of the Spanish community, there are references to ‘measures’ (18,625) and ‘prevention’ (17,927). Official bodies such as the ‘WHO’ (14,246) and ‘biosecurity’ (9303) are also mentioned. The ‘Delta’ variant (14,662) also appears in the public discourse. In English tweets, the same reference to the ‘Delta’ variant (21,969) and the concept of ‘wave’ (19,156) is observed. Other terms, such as ‘mask’ (16,668) and ‘deaths’ (18,805), are also present. The most peculiar elements are the references to ‘Africa’ (15,718) and ‘Ba2′ (16,668), the latter not used in any other language.

#### 3.2.3. Sentiment Analysis

For the Omicron data, a similar popularity trend to the previous case is observed. The absolute maximum is found at the beginning of the period, around November 2021, and decreases sharply after December and January 2021. There is a decrease in activity on the subject, but with successive upturns that represent relative peaks. From March 2022 onwards, the theme loses much popularity compared with the previous periods.

**Figure 10:**
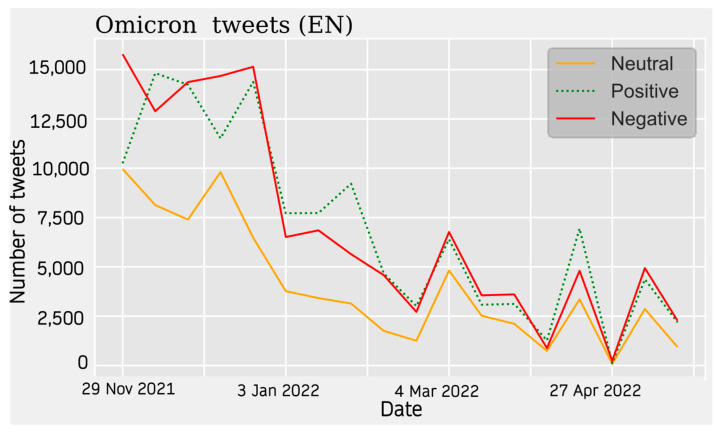
English Sentiment Analysis.

The predominant sentiment of opinion is negative. For tweets in Spanish, the distribution is very balanced between the three sentiment types, being evenly distributed throughout the listening period. At the beginning of the year 2022, a maximum of positive and neutral opinions are observed, along with a lower value of negative opinions. The sentiment of the hotspots is not very polarized in this case (see Figure 10).

**Figure 11:**
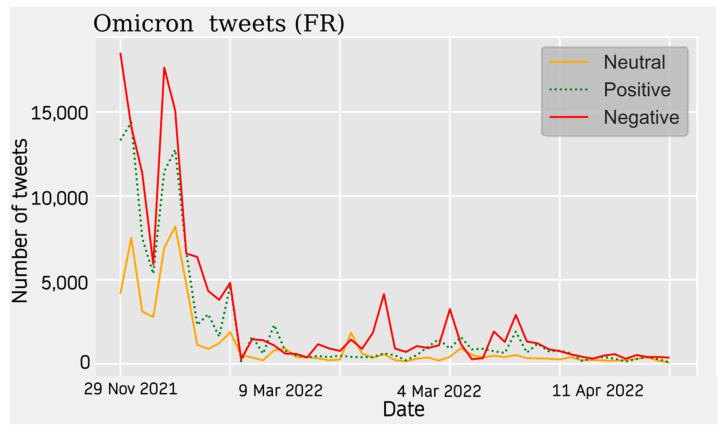
French Sentiment Analysis.

**Figure 12:**
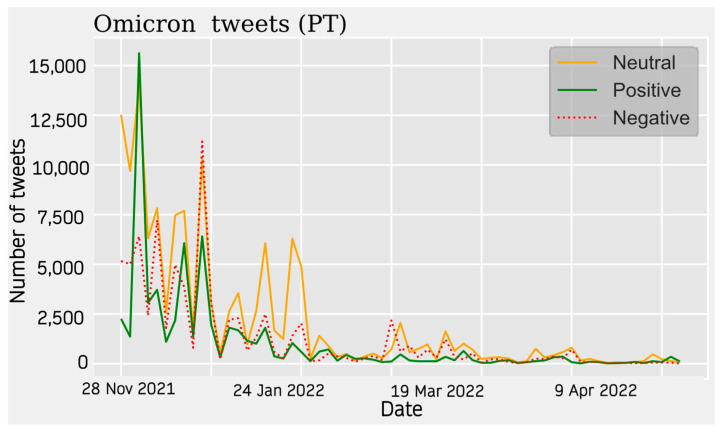
Portuguese Sentiment Analysis.

For the English database, a similar trend is observed, where the neutral opinion does not have as much weight, with more of the activity being balanced between positive and negative sentiment (Figure 11). In the French database, there are two peaks at the beginning of the study, with a clear negative presence, where neutral and positive opinions are considerably lower. For these data, activity ceases abruptly (Figure 12).

**Figure 13:**
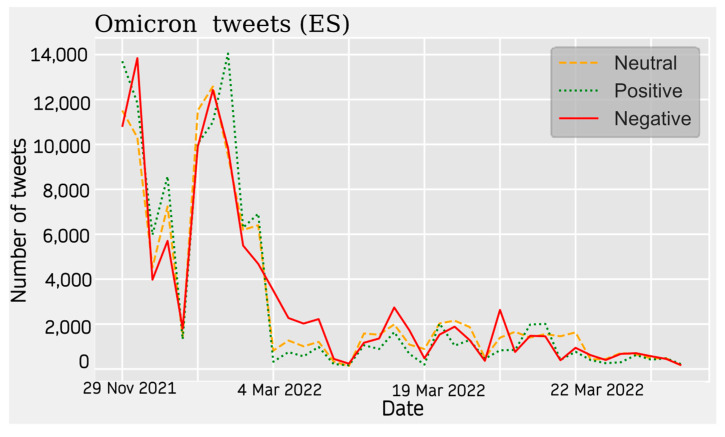
Spanish Sentiment Analysis.

In the case of tweets in Portuguese, we find a peak with a clear positive trend at the beginning of the listening period (November 2022), followed by a relative peak in negative opinion (December 2022). There was another spike in negative opinion in January 2022, and after this point, activity ceased (Figure 13).

**Figure 14:**
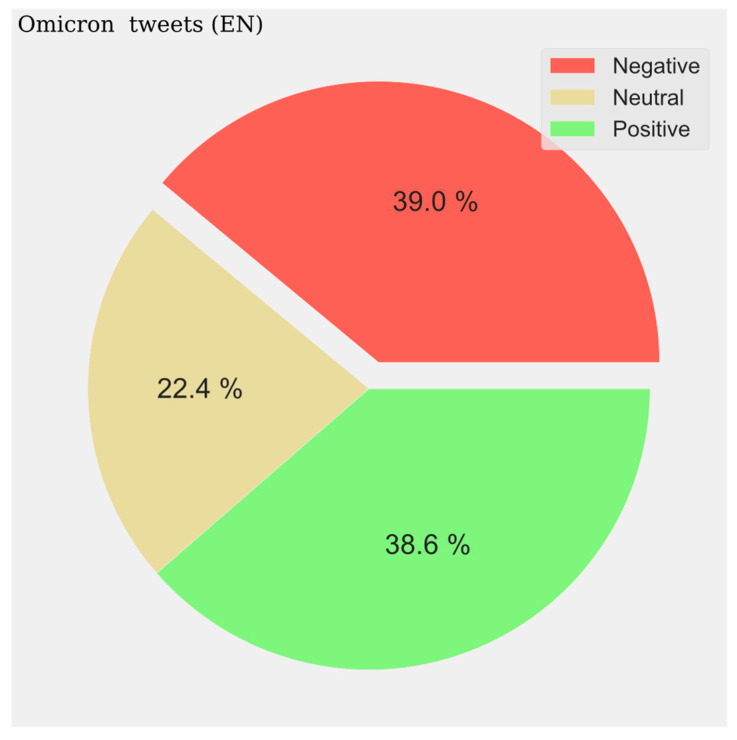
English Sentiment Analysis.

**Figure 15:**
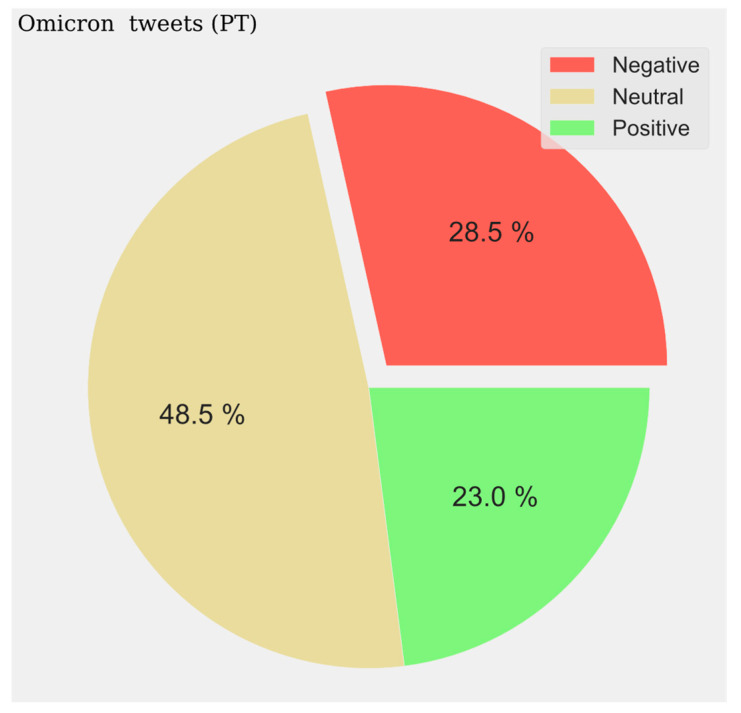
French Sentiment Analysis.

**Figure 16:**
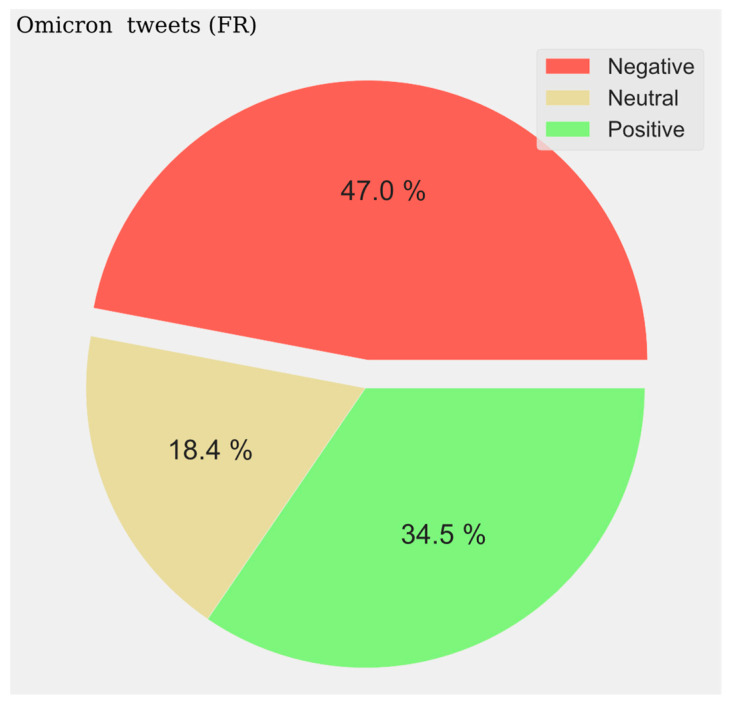
Portuguese Sentiment Analysis.

**Figure 17:**
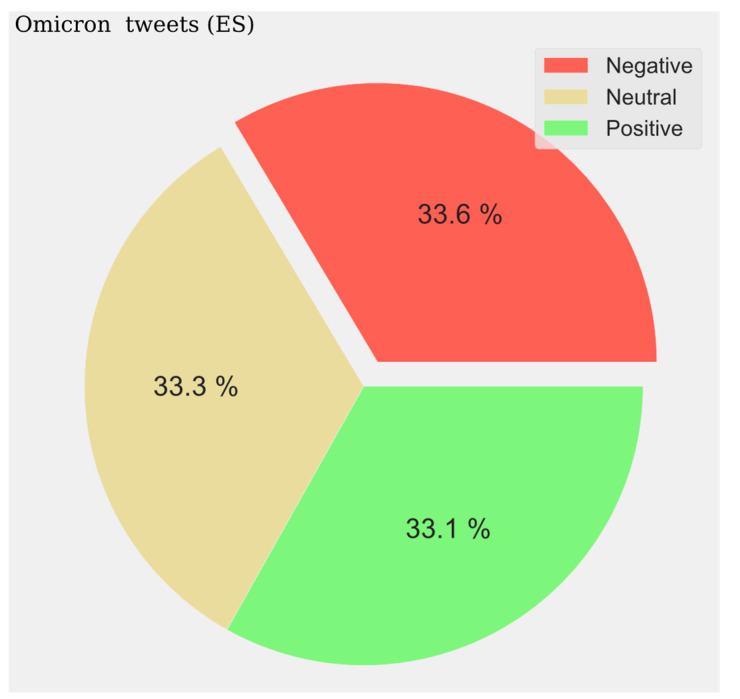
Spanish Sentiment Analysis.

The pie charts represent the distribution of positive, negative, and neutral opinions across all tweets regardless of period. A negative predominance is observed for Spanish (33.6%) (Figure 14), English (39%) (Figure 15), and French (47%) (Figure 16), with a very high level in the last case. A very low presence of neutral opinion is observed for French (18.4%) and English (22.4%). The opposite effect is observed for Portuguese content, where the neutral presence is very noticeable (48.5%) (Figure 17).

## 4. Discussion

The AstraZeneca vaccine crisis and the emergence of the Omicron variant were two critical events that shaped the pandemic management and impacted public discourse. This study aims to examine the key issues and controversies that dominated these two events. With a sample of 3,748,302 tweets in English, French, Portuguese, and Spanish, we provide a comprehensive representation of the public discourse during these crises. During the AstraZeneca COVID-19 vaccination crisis, the term “blood clot” was the most frequently used in all communities. However, unlike the Spanish and Portuguese communities, the English and French communities had a significant presence of tweets discussing the most severe risk category, “death”. Only the Portuguese tweets made direct references to a politician, specifically President Bolsonaro of Brazil. Additionally, the English and Spanish communities had more positive and neutral sentiments compared to the French community, which had the highest percentage of negative sentiment followed by the Portuguese community. Regarding the Omicron crisis, the public discourse focused on data and control measures, with a particular interest in cases and deaths, which had a high presence among the Spanish and Portuguese communities. Overall, the dominant sentiment towards the Omicron variant was negative, although the Spanish community showed a more balanced sentiment between negative, neutral, and positive.

### 4.1. The AstraZeneca Crisis

Our findings indicate the public may be more concerned about the potential for illness associated with this particular vaccine because there is a higher presence of the term “blood clot” in the four communities associated with the AstraZeneca vaccine compared to the other keywords searched. Although this concern, in the instance of a thrombus, does not correspond to the real risk, topics that people perceive to be a threat to their health tend to attract more public attention, reaching larger implications in their networks. According to the Pharmacovigilance Risk Assessment Committee (PRAC) of the European Centre for Disease Prevention and Control [30], the possibility of a person vaccinated with AstraZeneca developing a thrombus is highly rare.

Furthermore, if we compare the presence of the term ‘Blood clot’ among the four communities, the most striking case was the tweets in French, where the value is much higher, demonstrating the impact of the topic in this specific community. The term side effects also stand out more in the French-speaking community, with a value of almost double, compared to the other languages. France does not have a long history of anti-vaccine lobbying, as is the case in Great Britain [31]. In the 1990s, France faced controversies and resistance to vaccines for containing adjuvants [32], but a big change has occurred in the last 10 years when this country became one of the most resistant to vaccines in the world [18]. The French health authorities exercised significant pressure on those who were not vaccinated against COVID-19 by restricting access to areas that were at the time off-limits to those who fit into this category. Currently, 78% of the population has received the full course of COVID-19 vaccinations [33].

The word “deaths” is more frequently used in tweets from the English- and French-speaking communities, which further implies that there is serious concern about this vaccination given that it is associated with the most severe health risks conceivable. In the case of the Hispanic and Portuguese communities, references to deaths were much lower and did not seem to be a focus of interest related to the AstraZeneca vaccine for these users.

The two most frequent words that coincide with all four communities refer to the terms vaccines and Pfizer. Pfizer was the first pharmaceutical company to have its vaccine authorized for marketing in the European Union and the United States. If we look at how each community behaves, we can see that, in addition to Pfizer, other pharmaceutical companies have also been cited. The communities that cited them most in the public discourse on AstraZeneca were the Hispanic community and the Portuguese community. It is remarkable that when AstraZeneca is mentioned, reference is made to other pharmaceutical companies, possibly as a comparison between the vaccines of different companies. The discourse appears to be less polarized and more generalist among the Spanish- and English-speaking communities, with a focus on broader subjects such as immunization, booster shots, and health in general. With respect to the vaccine schedule, Spain has a history of achieving high immunization rates. This is also the case with the COVID-19 vaccination, with a complete schedule of 92.8% for those over 12 and a first booster dose of 55.1% [34].

The tweets in Portuguese cite scientific sources, such as the World Health Organization and Butantan, a Brazilian institute that produces the CoronaVac vaccine using ingredients from other countries, such as China. Following this same logic, the country also produces the AstraZeneca vaccine, but by the Oswaldo Cruz Foundation (Fiocruz) [35]. In addition, the words Brazil and Bolsonaro stand out in the tweets of the Portuguese-speaking community, a reference to the president of this country Jair Messias Bolsonaro (his term of office ended on 31 December 2022), being the only direct reference to a politician that appears in the whole analysis. Brazil is the country with the second highest number of deaths due to COVID-19, reaching on 25 November 2022, the mark of 669,665 thousand deaths [36]. The initial management of the health crisis was quite controversial, marked by strong politicization during the pandemic and rejection of science [37,38]. The Brazilian president has accumulated controversies, such as saying that COVID-19 was a “gripezinha” (little flu), defending herd immunity and the use of chloroquine as a preventive treatment against COVID-19 [37], even after the World Health Organization did not indicate this drug to prevent the disease [39]. Since the president has often claimed in the media that vaccines are ineffective while yet emphasizing their potential adverse effects, he has not received any vaccinations [37,40]. Another point to highlight is Bolsonaro’s frequent use of social media to communicate with his public and disseminate his ideas through this channel [41]. These may be a few of the causes behind this politician’s singular presence in the public discourse during the crisis.

The term “thrombus” stands out among the most often used terms in French, ranking fifth, along with the words ‘effects’ and ‘death’, confirming high-risk perception and consequently vaccine hesitancy. The French-speaking community appears to have quite strong opinions on the AstraZeneca vaccine, according to our findings. The low prevalence of neutral sentiment in this community demonstrates how strongly most individuals are positioned on this topic.

Across communities, sentiments on the AstraZeneca vaccination are typically polarizing. Our findings contrast with those made by Marcec and Likic [17] and Mahyoob et al. [28] who looked at tweets written in English about the same vaccine and found that sentiment in the Anglo-Saxon population is primarily negative, while our findings show that sentiment is predominantly positive, followed by negative and neutral sentiments. These two studies’ analyses, nevertheless, comprised a different time period a little earlier than the one used in our study. Concerning the Hispanic community, the sentiment was slightly similar, being more neutral, and followed by positive, and negative ones. The most negative sentiment was found in the French-speaking and Portuguese-speaking communities. In relation to this, it is commonly known that Twitter contains a significant amount of anti-vaccine lobbying. With these regards, efforts to fight anti-vaccination [42] must (a) closely collaborate with technological platforms to address anonymous anti-vaccine tweets; (b) concentrate efforts on misleading information; and (c) go beyond conventional factual methods, such as identifying, labeling, or eliminating fake news, to address the emotions brought on by personal memories, values, and beliefs.

In light of the findings from our study, it is crucial to provide a more comprehensive understanding of the impact of available information on vaccine acceptance. A previous study revealed strong knowledge and acceptance of COVID-19 vaccination among Italian undergraduates [43]. This highlights the effectiveness of the information strategy accompanying the national immunization campaign. It is noteworthy to mention that the perception of negative health consequences related to vaccines experienced a significant increase following the precautionary suspension of Vaxzevria, the AstraZeneca vaccine for COVID-19. With these regards, a more robust understanding of the broader impact of information on vaccine acceptance should be achieved, emphasizing the need for continued investigation and targeted interventions to address vaccine hesitancy.

### 4.2. The Omicron Crisis

According to our findings, risk-related terms, such as ‘infects’ and ‘deaths’, were used more frequently by users according to the word search, rather than ‘mask’ and ‘risks’. Since nobody knew if the Omicron strain could be as lethal as its predecessor, the Delta strain, which had previously predominated until then [10], the development of this variant happened at a time when there had already been 5 million deaths globally [36]. When an event poses an unknown risk, as in the case of the coronavirus initially and the omicron variant later, may provoke an emotional reaction to decision-making regarding this, risk perception is more impacted [13]. This phenomenon has also been reflected in our data since the perception of risk and fear has a strong relationship with the emergence of the new variant. In this sense, people’s interest in focusing on these subjects may be an indication that their perception of risk may be closer to the real risk, which is becoming infected. In this sense, perceiving this strain as a risk to their health could lead people to take attitudes towards prevention [12], such as getting vaccinated, and using masks, among others.

We have identified the 16 most frequent words in each community. The first place is taken by the word ‘variant’, present in the four communities and cited more than 300 thousand times. There are others related to this term, such as ‘Delta’, ‘virus’, ‘Ba2′, ‘infection’, and ‘cases’. The word ‘vaccine’ stands out as the second most used word in the Portuguese and French-speaking communities, with more than 100 thousand citations. Other terms in the four languages related to the vaccine are also observed, such as ‘dose’, ‘immunity’, ‘vaccinated’, ‘Pfizer’, ‘booster’, and ‘rate’. These references may be connected to the fact that Omicron’s arrival left the public uncertain as to whether vaccines would continue to protect against the new variety, despite the WHO’s repeated statements that vaccines play an important role in preventing serious diseases and deaths [10].

The Hispanic case is highlighted by several references to preventive measures to avoid the spread of the virus. From the public health point of view, prevention seeks to ensure the protection of diseases, reducing their incidence and prevalence in the population [44]. Spain was one of the countries that have maintained some preventive measures against COVID-19 for a long period, such as the use of masks indoors when other European countries had already relaxed these rules [45].

It is worth noting that, in general, the discourse on Omicron is quite generalized in the four communities, with specific mentions to Africa, related to the fact that omicron was identified for the first time in this continent, and also references to the pharmaceutical company Pfizer. In relation to scientific institutions, the only one that was highly cited was the WHO in the Hispanic and Portuguese cases, showing the importance of this international organization in the public discourse during health crises.

Sentiments towards Omicron in the Spanish, English, and French communities are predominantly negative, i.e., the perception that this variant represents a risk to users is high. Our results coincide with those found by [29], when analyzing tweets in several languages, they found quite negative sentiments related to this variant. On the other hand, in our study, the case of the Portuguese community was dominated by neutral sentiment similar to those found by [30], showing that 50.5% of the posts were neutral, the other emotions being composed of sentiments such as ‘bad’, ‘good’, ‘terrible’ and ‘great’. Finally, it is important to note that a previous study concluded that vaccination plays a key role in dropping the negativity of people, thus promoting their psychological well-being [46].

### 4.3. Limitations

This study has some limitations. Our analysis did not allow us to identify geographically the origin of all users, which limited us in terms of analyzing the data taking into account the socio-health contexts of these countries, except in cases where it was possible to detect specific references to countries in the posts published. However, we consider that this strategy also has its strength in the sense that people, by sharing a language, form a virtual community in which topics of interest are discussed. In addition, although our search has completed four languages covering several countries, it is quite concentrated in some regions, for this reason, we consider that in future research it would be relevant to broaden this horizon to include other languages in order to ensure more diversity. The fact that our paper could only analyze text and not visual content was another limitation of the analysis. We recommend further research on this topic given the importance of visuals during health crises [47,48].

The purpose of using automatic techniques is the possibility of obtaining an overall balance of all the data, where it would be impossible to carry out an individual evaluation. This procedure evaluates a concept subject to a certain subjectivity, which is why we have used these more quantitative results obtained previously. Conducting a sentiment study and applying it to independent conclusions could lead to inaccurate results. This methodology, in isolation, may have limitations, which is why this study has been combined with the previous analyses, allowing global conclusions to be drawn from the data analyzed.

Finally, although we have not carried out a detailed analysis of the topics investigated, we want to highlight the fact that working with a large volume of data has allowed us to draw a general overview of the perception of risks related to the events investigated. With these regards, our paper contributes to knowledge in four ways. First, it broadens the scope of crisis communication by revealing social media’s role in the constructs of public discourse. Second, we suggest that social media is the optimal place to explore risk perception during a health crisis. Two case studies were used to support this claim. Third, we stress the cultural diversity amongst global communities, demonstrating the necessity of tailoring crisis communication strategies to social and cultural traits. Fourth, while our study does not delve extensively into the nuanced interpretation of public sentiment, we believe that it lays the foundation for future research and provides valuable insights into the role of social media in shaping public discourse during health crises. By uncovering the constructs and patterns of public discourse, we contribute to the broader field of crisis communication.

## 5. Conclusions

The terms “Blood Clot” and “deaths” have been often used in tweets about the AstraZeneca crisis to express concerns about the impact the vaccine would have on the population, with a particular emphasis on the French population. In this way, the public discourse shows how the perceived risk associated with this vaccine is highly exaggerated given how unlikely it is that these side events would occur. However, as has happened in several countries where AstraZeneca has seen significant widespread criticism, this idea linking the vaccination to risk may make individuals hesitant to receive this immunizer. In the case of Omicron, the public discourse demonstrates how risk perception tends to be closer to real risk given that infections and deaths have received significant public attention. A decision favoring prevention may have been influenced by the perception that this variant might increase the number of cases. In this sense, the public discourse of Omicron was largely negative as it was perceived as a threat to their health. The main contribution of this paper highlights that these two crises have shown that public discourse during health crises might lead to different behaviours. While public discourse about AstraZeneca might enhance the reduction of preventive measures by reducing the number of vaccinated population, the Omicron discourse could increase preventive actions such as the use of masks to prevent infections.

## Figures and Tables

**Table 1:** Database properties.

Dataset	N° Tweets	Start	Period End	Total [Days]
AstraZeneca English	720,717	20 April 2021	29 April 2022	374
AstraZeneca French	444,244	19 April 2021	29 April 2022	375
AstraZeneca Portuguese	550,895	14 April 2021	29 April 2022	380
AstraZeneca Spanish	778,479	20 April 2021	29 April 2022	374
Omicron English	323,809	29 November 2021	29 April 2022	151
Omicron French	318,961	29 November 2021	29 April 2022	151
Omicron Portuguese	274,726	29 November 2021	29 April 2022	152
Omicron Spanish	336,471	29 November 2021	29 April 2022	151
Total	3,748,302			

**Table 2:** Keywords for topics studies in AstraZeneca Tweets.

English	French	Portuguese	Spanish
‘thrombus, blood clot’	‘caillot’	‘trombose, trombo’	‘trombos, coágulos’
‘side effects’	‘effets secondaires’	‘efeitos colaterais’	‘efectos secundarios’
‘anti-vax’	‘anti-vax’	‘anti-vax’	‘anti vax’
‘deaths’	‘décédé’	‘mortos, mortes’	‘muertos, fallecidos’
‘lies, fake’	‘menteurs, données fausses’	‘mentiras, dados falsos, engano’	‘fake, falsos, mentiras’

In addition to the terms used, their derivative words (plural and singular) have been applied.

**Table 3:** Keywords for topics studies in Omicron Tweets.

English	French	Portuguese	Spanish
‘mask’	‘masque’	‘máscara’	‘mascarilla’
‘infections, cases’	‘infecté, cas’	‘infecções, casos’	‘infecciones, contagios’
‘deaths, die’	‘décédé’	‘mortos, mortes’	‘muertes, fallecidos’
‘risks, danger’	‘risque, péligro’	‘risco, peligro’	‘riesgo, peligro’

In addition to the terms used, their derivative words (plural and singular) have been applied.

**Table 4:** Importance of topics in the AstraZeneca crisis.

	EN		FR		PT		ES		Max
	Tweets	Norm. max.V	Tweets	Normal max.V	Tweets	Normal max.V	Tweets	Normal max.V	Percentage [%]
Blood Clot	47,322	0.48	61,176	1	17,437	0.23	22,525	0.21	13.77%
Side Effects	10,978	0.46	14,588	1	10,677	0.59	8142	0.32	3.28%
Anti-Vaxx	11,113	1	1635	0.24	552	0.06	2042	0.17	1.54%
Deaths	75,418	1	41,046	0.88	8727	0.15	14,568	0.18	10.46%
Lies	18,152	1	2545	0.23	2705	0.19	1108	0.06	2.52%

**Table 5:** The word results in each community related to the AstraZeneca crisis.

*n*	English	*n*	French	*n*	Portuguese	*n*	Spanish
499,232	vaccine	261,791	vaccines	184,006	vaccine	398,072	vaccines
161,858	Pfizer	129,594	Pfizer	100,414	Pfizer	115,731	Pfizer
60,031	India	96,172	Moderna	58,378	Oxford	83,869	vaccination
59,716	Moderna	65,505	vaccination	36,681	Brazil	46,895	Moderna
58,929	jab	45,641	thrombosis	34,754	Covax	41,840	booster
45,971	vaccination	33,268	vaccinate	34,059	health	36,749	virus
45,126	Oxford	32,111	France	28,524	Moderna	32,527	seniors
44,872	UK	31,581	Janssen	27,320	Bolsonaro	29,654	government
43,886	case	25,004	Johnson	24,962	vaccination	29,470	health
42,837	blood	23,504	vaccinated	22,369	Butantan	23,912	Argentina
41,397	death	21,871	effects	20,748	effects	23,202	Janssen
38,162	clot	21,473	report	20,663	OMS	22,939	Covax
34,725	study	21,077	French	19,699	study	22,491	Sinovac
34,451	health	20,656	injection	19,207	reaction	20,362	Sputnik
34,043	vaccinated	19,776	death	19,003	Bharat	19,706	vaccinated
32,892	government	19,422	health	18,975	Johnson	19,495	Sinopharm
30,813	booster	16,123	variant	20,594	Sputnik	19,146	case
29,826	effective	15,234	risk	18,023	deprived	18,053	Oxford

**Table 6:** Importance of topics in the Omicron crisis.

	EN		FR		PT		ES		Max
	Tweets	Norm. max.V	Tweets	Norm. max.V	Tweets	Norm. max.V	Tweets	Norm. max.V	Percentage [%]
Mask	20,435	1	12,861	0.64	15,529	0.90	9843	0.46	6.31%
Infected	63,427	0.77	58,593	0.72	68,824	0.98	86,142	1	25.60%
Deaths	28,263	1	24,455	0.88	9476	0.40	21,057	0.72	8.73%
Risk	13,447	0.98	13,548	1	10,108	0.87	12,672	0.89	4.25%

**Table 7:** The words result in each speaking community related to the Omicron crisis.

*n*	English	*n*	French	*n*	Portuguese	*n*	Spanish
78,643	variant	92,819	Variant	56,772	Variant	127,381	variant
54,516	case	61,142	Vaccine	48,064	Vaccine	51,199	virus
21,969	Delta	29,040	Dose	45,688	Cases	37,318	cases
21,584	virus	27,443	France	23,894	Pandemic	29,690	dose
19,156	wave	27,259	Delta	20,222	Brazil	27,746	pandemic
19,038	health	23,058	Virus	19,459	Dose	21,791	vaccine
18,805	death	20,756	Vaccination	17,436	Immunity	18,625	measures
18,624	south	16,775	Pfizer	16,925	Children	17,927	prevention
18,469	UK	16,607	Messages	16,896	Health	16,949	vaccines
18,440	infection	14,311	Children	15,572	Vaccinated	14,662	Delta
17,639	booster	13,679	Health	15,568	Pfizer	14,246	OMS
16,668	Ba.2	13,132	Deaths	11,681	Virus	14,203	health
16,636	mask	12.998	Mask	11,361	Africa	12,385	important
15,718	Africa	12,214	Rheum	10,615	OMS	11,467	symptoms
13,928	vaccinated	11,669	Biotech	10,422	Influence	11,408	variants
11,301	government	10,197	Ba.2	9415	data	10,571	Pfizer

## Data Availability

Data are available through the corresponding author upon reasonable request.
